# Hepatic Fever

**Published:** 1890-05-17

**Authors:** 


### THE PRACTITIONER'S MIRROR AND RETROSPECT.

[*The Editor will be glad to receive offers of co-operation and contributions from members of the profession. There is no doubt that much valuable material full of interest and instruction is at present lost to science. This is largely so in the case of* (1) *the younger and less-known members of the hospital staff* (2) *those connected with cottage hospitals, and* (3) *the great body of medical practitioners not attached to any institution. The co-operation of all is cordially invited to enable the Editor to make* THE HOSPITAL *of the utmost possible use and value to the profession. Specialists willing to review books on their special subjects are invited to communicate this fact at once. All letters should be addressed to* THE EDITOR, THE LODGE, PORCHESTER SQUARE, LONDON, W.]

### MEDICINE.

### HEPATIC FEVER.

It was in Holland that a French general, writing to the Emperor Napoleon, and complaining of the destruction of the garrison by fever, received a reply, "L' homme meurt partout." Perhaps the term fever was too commonplace to enlist the sympathy of the great general, although even at that time the ingenuity of nosologists had invented various high-sounding, and, to ignorant people at least, more terrifying names. Fevers have been so differentiated on paper that it is exceptional to meet with a case resembling the type. So much is this the case, especially with tropical fevers, that authors have invented compound names, such as typho-malarial, malario-typhoid, &c. And other authors have referred hybrid fevers, not susceptible of classification by symptoms under any head, to such divisions as "climatic fever" and "undefined fever." If we take remittent fever, for instance, we find that it has been described under at least forty different names, either in accordance with the locality, or in accordance with peculiar symptoms which may have developed. Thus we have "jungle fever," "terrae fever," "Bengal fever," &c.; and "bilious remittent," "gastric remittent," "adynamic remittent," &c. It is much the same with regard to enteric fever. Da Costa states that many divisions are uncalled for, remarking "nature herself, by the readiness with which she permits essential traits to be interchanged, or to become blended on the same attack, proves that even groups are not widely distinct. The classification of fevers is, to a great extent, matter of speculation." Still new names are invented, most frequently from a Greek base. A recent author has just invented a new name as above, but has not added to, as we think, unnecessary labour by using unaccustomed terms.

Dr. William Pepper, of Philadelphia, has recently published an article headed "Hepatic Fever" (*Philadelphia Medical News*, March). He does not speak of the ordinary fever which attends acute hepatic affections, as in acute catarrhal jaundice, or following the passage of a gall stone. But he speaks of a distinctly paroxysmal fever at regular or irregular intervals, after which jaundice often makes its appearance. At the time of occurrence of this paroxysmal fever there may, or may not be, pain in the region of the liver. But sometimes the pain is so severe that it appears as if a calculus were passing. From recurring paroxysms anæmia is quickly developed. Fever, Dr. Pepper states, comes very often as a septic process, from absorption into the system of some morbid pyrogenic poisonous substance. But it may arise from a disorder of the nervous apparatus controlling heat production and dissipation, and this disorder may be either centric directly, or may be induced by reflex irritation. The liver, besides its bile-secreting function, possesses the power of arresting pyrogenic substances, and of destroying their morbid properties, so that, granting the functional activity of the liver, pyrogenic substances and ptomaines are not able to penetrate the system and generate pyrexia. If there is a condition of the liver where the bile-ducts are distended with accumulated bile, the ptomaine-destroying function of the liver is more or less paralysed, and there will pass into the system poisonous substances which may excite fever. But Dr. Pepper confesses that the explanation of the periodicity which occurs in cases of hepatic fever is attended with difficulty. If there is a fixed lesion, as, for instance, a suppurative inflammation of the gall bladder, there we have a distinct pus-forming centre, and we may have a true pyæmia. But in those cases where the paroxysms do not recur every day, or with any regularity, "it seems as if we must assume the existence of a lesion, not continually sufficient to excite fever, but which is extremely liable to be increased by external causes." We confess that we do not quite understand this, for first the so-called hepatic fever is attributed to absorption of ptomaines, and then some lesion is regarded as a necessity.

Now, some of the cases mentioned by Professor Pepper bear a strong resemblance to what we have been accustomed to regard as Relapsing Fever. Relapsing fever is characterized by a tendency to recur at intervals, and jaundice is often a prominent symptom, sometimes occurring suddenly, at other times gradually. There may be intense jaundice, and also black vomits, which has been described as congestive or bilious relapsing fever. Other cases mentioned by the author lead to the suspicion of gall stone. Yet we believe that the liver is but secondarily implicated by the congestion attending the onset of the fever, or a gall stone, and that the condition of the liver is not the original cause of the fever. Vandyke Carter referred relapsing fever to a spirillum in the blood, which, it is stated, very much resembles a worm known as *spirillum plicatile*, sometimes found in water. The only fever which, as we think, can be properly regarded as hepatic fever is that which attends the formation and progress of deep-seated insidious liver abscess. But this is not a fever marked by sudden rigors and the usual hot and sweating stage. It is a fever which occurs at irregular intervals, and is sometimes so slight that it is not perceptible until the clinical thermometer is used. Nevertheless, it is very suggestive, and may be the only symptom of a deep-seated hepatic abscess. Eventually it may develop into hectic; or it may subside, if, as occasionally happens, the liver abscess becomes quiescent, being converted into a cheesy substance; or as there is reason to believe sometimes occurs, the abscess i absorbed.

